# OrfX, a Nucleomodulin Required for *Listeria monocytogenes* Virulence

**DOI:** 10.1128/mBio.01550-17

**Published:** 2017-10-31

**Authors:** Andrzej Prokop, Edith Gouin, Véronique Villiers, Marie-Anne Nahori, Renaud Vincentelli, Mélodie Duval, Pascale Cossart, Olivier Dussurget

**Affiliations:** a Institut Pasteur, Unité des Interactions Bactéries-Cellules, Paris, France; b Inserm, U604, Paris, France; c INRA, USC2020, Paris, France; d Université Paris Diderot, Sorbonne Paris Cité, Paris, France; e CNRS, Aix Marseille Université, UMR7257, AFMB, Marseille, France; University of California, San Francisco

**Keywords:** *Listeria monocytogenes*, nucleomodulin, pathogenesis, RybP, oxidative burst

## Abstract

*Listeria monocytogenes* is a bacterial pathogen causing severe foodborne infections in humans and animals. *Listeria* can enter into host cells and survive and multiply therein, due to an arsenal of virulence determinants encoded in different loci on the chromosome. Several key *Listeria* virulence genes are clustered in *Listeria* pathogenicity island 1. This important locus also contains *orfX* (*lmo0206*), a gene of unknown function. Here, we found that OrfX is a small, secreted protein whose expression is positively regulated by PrfA, the major transcriptional activator of *Listeria* virulence genes. We provide evidence that OrfX is a virulence factor that dampens the oxidative response of infected macrophages, which contributes to intracellular survival of bacteria. OrfX is targeted to the nucleus and interacts with the regulatory protein RybP. We show that in macrophages, the expression of OrfX decreases the level of RybP, which controls cellular infection. Collectively, these data reveal that *Listeria* targets RybP and evades macrophage oxidative stress for efficient infection. Altogether, OrfX is after LntA, the second virulence factor acting directly in the nucleus.

## IMPORTANCE

*Listeria monocytogenes* is a model bacterium that has been successfully used over the last 30 years to refine our understanding of the molecular, cellular, and tissular mechanisms of microbial pathogenesis. The major virulence factors of pathogenic *Listeria* species are located on a single chromosomal locus. Here, we report that the last gene of this locus encodes a small secreted nucleomodulin, OrfX, that is required for bacterial survival within macrophages and in the infected host. This work demonstrates that the production of OrfX contributes to limiting the host innate immune response by dampening the oxidative response of macrophages. We also identify a target of OrfX, RybP, which is an essential pleiotropic regulatory protein of the cell, and uncover its role in host defense. Our data reinforce the view that the secretion of nucleomodulins is an important strategy used by microbial pathogens to promote infection.

## INTRODUCTION

*Listeria monocytogenes* is a facultative intracellular pathogen responsible for listeriosis, a food-borne disease in humans and animals. Its clinical manifestations range from self-limiting febrile gastroenteritis in healthy persons to fetal infections with up to 80% mortality (1) and life-threatening septicemia and meningitis in neonates and elderly and immunocompromised individuals (2). Its pathogenesis relies on the production of virulence factors that are instrumental in crossing host barriers, escaping immunity, reprogramming host cell genes, and ultimately, replicating within host cells (3). Upon infection of the host, *L. monocytogenes* can invade multiple cell types, including macrophages (4). Once internalized, bacteria escape from the internalization vacuole by secreting the pore-forming toxin listeriolysin O (LLO) and the two phospholipases PlcA and PlcB (5–7). PlcB is a lecithinase that undergoes maturation by proteolytic cleavage mediated by the metalloprotease Mpl (5, 8). When bacteria reach the cytosol, they rapidly replicate and produce the surface-associated protein ActA, which triggers actin-based motility, contributing to cell-to-cell spread (9). The expression of all these factors is regulated positively by the transcriptional factor PrfA (5, 10, 11). Inactivation of LLO, ActA, or PrfA leads to severe attenuation of *Listeria* virulence (5, 9, 11, 12), while mutants that do not produce PlcA, PlcB, or Mpl exhibit a milder decrease of virulence in mouse models of infection (13). Strikingly, the genes encoding PrfA and the virulence factors necessary for intracellular survival are located on a single 9-kb locus of the *L. monocytogenes* chromosome, known as the virulence gene cluster or *Listeria* pathogenicity island 1 (4, 14). The other pathogenic *Listeria* species, *Listeria ivanovii*, carries a copy of this locus (4). In contrast, this genomic island is partially or totally absent from the genomes of nonpathogenic *Listeria* species, with the exceptions of *Listeria seeligeri* (15) and some atypical strains of *Listeria innocua* (16, 17), in which it is considered a remnant of an ancestral *L. monocytogenes* island. In addition to the above-described well-characterized virulence genes, the virulence locus carries at its 3′ end a small open reading frame, *orfX* (*lmo0206*), which encodes a 107-amino acid (aa) putative protein without any ortholog in other species or any conserved domain except an N-terminal secretion signal sequence (18). OrfX remains the only member of the virulence locus that is of unknown function. Interestingly, like that of most members of the locus, the expression of *orfX* is low in rich broth medium and highly induced in human blood, suggesting a putative role of this gene in *Listeria*’s intracellular lifestyle (19). In agreement with this hypothesis, the expression of *orfX* has been shown to be induced within host cells (20). More recently, a study confirmed this induction in four *L. monocytogenes* strains belonging to the three major lineages (21).

The Ring1 YY1-binding protein RybP is a zinc finger protein that regulates gene expression at the transcriptional level by interacting with the Polycomb complex and acts as an adaptor to mediate protein-protein interactions (22). It is conserved in vertebrates and plays a vital role in embryonic development. RybP promotes gene silencing and maintains transcriptional repression of important developmental genes (22). It is also part of the BCL6 corepressor (BCOR) complex (23) and mediates specific interactions between the E2F and YY1 transcription factors to activate the Cdc6 promoter and DNA replication (24). Furthermore, RybP binds to E3 ligase MDM2, preventing proteasomal degradation of the tumor suppressor p53 (25). Importantly, RybP has been shown to promote apoptosis by interacting with proteins such as procaspase-8, procaspase-10, Fas-associated death domain (FADD), Hippi, fibronectin type III and ankyrin repeat domains 1 (FANK1), and the viral protein apoptin (26–29). Besides its essential developmental, proapoptotic, and antioncogenic roles, RybP has recently been identified as a regulator of the innate immune response in *Drosophila* (30).

Due to the importance of the virulence locus in *Listeria* pathogenesis, we investigated the role of OrfX. Here, we report that *orfX*, like the other genes of the locus, is required for *L. monocytogenes* virulence in mice. Bacteria expressing OrfX dampened the macrophage oxidative response to infection, which contributed to intracellular survival in these cells. We further demonstrate that OrfX is secreted and reaches the host cell nucleus, where it targets RybP. The expression of OrfX reduces the levels of RybP, which controls cell infection. Thus, OrfX is a new member of the growing family of nucleomodulins that protect bacteria from host cell defenses during infection.

## RESULTS

### OrfX is a virulence factor.

**FIG 1:**
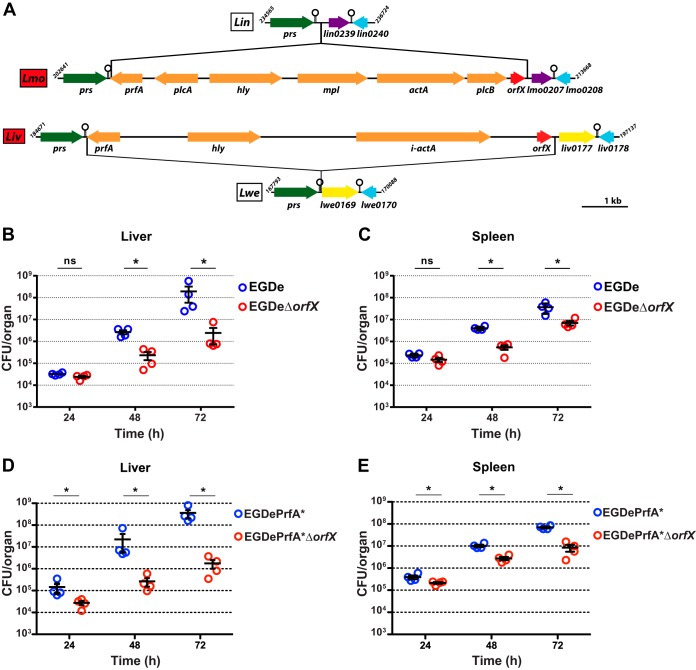
*Listeria monocytogenes* OrfX is a virulence factor. (A) Genomic organization of the *orfX* locus in *Listeria* species. Data from the complete genome sequences of nonpathogenic *L. innocua* CLIP11262 (*Lin*) and *L. welshimeri* SLCC5334 (*Lwe*) and pathogenic *L. monocytogenes* EGDe (*Lmo*) and *L. ivanovii* PAM55 (*Liv*) were used to map the genes (arrows) and the putative terminators (hairpins). Green, purple, blue, and yellow arrows represent orthologs. Orange arrows represent genes of the virulence cluster. Red arrows represent the *orfX* gene. (B to E) Multiplication of *L. monocytogenes* EGDe and EGDe*ΔorfX* (B and C) and EGDePrfA* and EGDePrfA*ΔorfX (D and E) strains in livers (B and D) and spleens (C and E) of BALB/c mice challenged by intravenous injection of a sublethal infectious dose (10^4^ bacteria/mouse). For each strain, CFU were determined in organs of four mice at 24, 48, and 72 h postinfection. Statistical analysis was performed using the Student two-tailed *t* test. Mean values with *P* values of ≤0.05 (*) were considered statistically different.

The *orfX* gene (*lmo0206*) belongs to the group of *L. monocytogenes* genes that are absent from the genome of the nonpathogenic species *L. innocua* (31). Comparative genomic analysis showed that *orfX* is also present in the virulence gene cluster of the pathogenic species *L. ivanovii* but absent from the nonpathogenic species *Listeria welshimeri* (Fig. 1A). Therefore, *orfX* was an attractive candidate virulence gene.

In order to study the contribution of OrfX to the infectious process both *in vitro* and *in vivo*, we created an *orfX* deletion mutant in the *L. monocytogenes* strain EGDe. We first addressed the role of OrfX *in vivo*. BALB/c mice were challenged intravenously with the wild-type EGDe or the EGDeΔ*orfX* mutant strain, and the numbers of bacteria in *Listeria* primary target organs, i.e., the liver and spleen, were determined 24, 48, and 72 h postinfection. The numbers of mutant and wild-type bacteria recovered in both organs were similar 24 h postinfection. The attenuation of the mutant became significant 48 h postinfection and even higher 72 h postinfection. At the latter time point, inactivation of *orfX* resulted in a 100-fold decrease in the bacterial load in the liver (Fig. 1B) and a 10-fold decrease in the spleen (Fig. 1C). Inactivation of *orfX* in a second strain, *L. monocytogenes* EGDePrfA*, whose mutation in PrfA causes constitutive overexpression of virulence factors (32), decreased the bacterial burdens in livers and spleens of infected mice even further (Fig. 1D and E). Together, these results establish an important role for OrfX in *Listeria* virulence.

### OrfX contributes to *Listeria* multiplication and dampens the oxidative response in macrophages.

**FIG 2:**
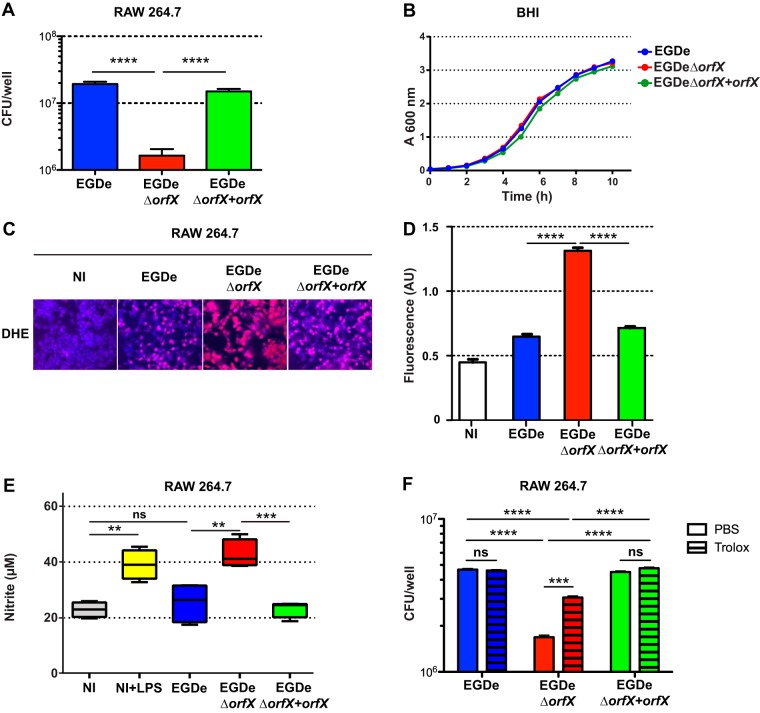
OrfX promotes *Listeria* survival and dampens the oxidative response in macrophages. (A) Effect of the *orfX* deletion on *L. monocytogenes* intracellular survival in macrophages. RAW 264.7 macrophages were infected at an MOI of 10 with the wild-type strain (EGDe), the mutant strain (EGDeΔ*orfX*), or the complemented strain (EGDeΔ*orfX*+*orfX*). The number of CFU was determined 8 h after the addition of gentamicin. Statistical analysis was performed using the Student two-tailed *t* test. Mean values are expressed as CFU/well ± SEM. ****, *P* ≤ 0.001. (B) Deletion of *orfX* does not affect *Listeria monocytogenes*’ growth in BHI. Growth of the wild-type (EGDe), mutant (EGDeΔ*orfX*), or complemented (EGDeΔ*orfX*+*orfX*) strain was measured in BHI at 37°C, 200 rpm, and 21% O_2_ atmosphere. Mean values are expressed as the measure of *A*_600_ ± SEM (*n* = 3). (C and D) OrfX inhibits superoxide production by macrophages. (C) RAW 246.7 macrophages were left uninfected (NI) or infected by the *L. monocytogenes* EGDe, EGDeΔ*orfX*, or EGDeΔ*orfX*+*orfX* strain for 24 h. Cells were stained with 5 µM dihydroethidium (DHE) and analyzed by fluorescence microscopy. Representative images are shown. DHE exhibits blue fluorescence until it is oxidized and stains the nucleus fluorescent red, indicating superoxide production. (D) Quantification of the relative superoxide levels. Superoxide levels are represented as the ratios of oxidized to nonoxidized DHE fluorescence intensities shown in panel C. Statistical analysis was performed using the Student two-tailed *t* test. Mean values with *P* values of ≤0.05 were considered statistically different. ****, *P* ≤ 0.0001. (E) OrfX inhibits NO production. RAW 246.7 macrophages were infected by the *L. monocytogenes* wild-type (EGDe), mutant (EGDeΔ*orfX*), or complemented (EGDeΔ*orfX*+*orfX*) strain or treated with 1 µg/ml LPS for 24 h. NO production was measured using the Griess reagent. Statistical analysis was performed using the Student two-tailed *t* test. Mean values with *P* values of ≤0.05 were considered statistically different. **, *P* ≤ 0.01; ***, *P* ≤ 0.001; ns, nonsignificant difference. (F) Inhibition of oxidative response by OrfX contributes to *Listeria* survival in macrophages. RAW 264.7 macrophages treated or not with 50 μM trolox were infected at an MOI of 20 with the wild-type strain (EGDe), the mutant strain (EGDeΔ*orfX*), or the complemented strain (EGDeΔ*orfX*+*orfX*). The number of CFU was determined 8 h after the addition of gentamicin. Statistical analysis was performed using the Student two-tailed *t* test. Mean values are expressed as CFU/well ± SEM. ***, *P* ≤ 0.001; ****, *P* ≤ 0.001.

Since macrophages are important for the control of *L. monocytogenes* infection, we examined the role of OrfX in RAW 264.7 macrophages. Cells were infected with the wild-type EGDe strain, the EGDeΔ*orfX* mutant, or a complemented EGDeΔ*orfX* strain producing OrfX (EGDeΔ*orfX*+*orfX*), and the numbers of intracellular bacteria were determined 8 h postinfection. The number of Δ*orfX* mutant bacteria was significantly reduced compared to that of the wild-type strain in macrophages (Fig. 2A), while the deletion mutant grew as well as the wild type in broth media, such as brain heart infusion (BHI) (Fig. 2B). The wild-type and complemented strains had similar multiplication rates in macrophages, providing evidence that *orfX* was the sole factor responsible for the growth defect phenotype of the mutant.

We next assessed whether OrfX affects the capacity of the cell to regulate oxidative pathways, which are central to the control of microbial pathogens by phagocytes (33). We examined the level of reactive oxygen species production in macrophages infected with the wild-type EGDe, EGDe*ΔorfX*, or complemented strain. The superoxide level was determined by using dihydroethidium dye (34), which emits red fluorescence upon oxidation by superoxide anions, as shown by the images in Fig. 2C. Noninfected cells had low levels of red fluorescence, corresponding to the basal level of superoxide production at homeostasis (Fig. 2C, first panel, and D). Interestingly, infection with the wild-type EGDe strain or the complemented EGDeΔ*orfX*+*orfX* strain did not lead to any major superoxide burst (Fig. 2C, second and fourth panels, and D). In contrast, macrophages infected with the *ΔorfX* mutant produced a large amount of superoxide anions (Fig. 2C, third panel), which was much higher than that produced by noninfected macrophages or by macrophages infected with wild-type and complemented strains (Fig. 2D). We next investigated nitric oxide (NO) production in infected macrophages using the Griess diazotization reagent and a sodium nitrite standard curve. Infection with the wild-type EGDe strain did not lead to increased NO production by macrophages (Fig. 2E). In contrast, infection with the Δ*orfX* strain showed increased production of NO compared to that in noninfected macrophages or macrophages infected with the wild-type or the complemented strain (Fig. 2E). This NO burst was similar to that induced by lipopolysaccharide (LPS) treatment (Fig. 2E). These results suggest that OrfX dampens the oxidative response of macrophages to infection.

To assess whether the decreased survival of the *ΔorfX* strain was due to the increased oxidative response of macrophages, we treated the cells with the antioxidant trolox before infection with the wild-type EGDe strain, the EGDeΔ*orfX* mutant, or the complemented EGDeΔ*orfX* strain producing OrfX, and the numbers of intracellular bacteria were determined 8 h postinfection. The number of Δ*orfX* mutant bacteria was significantly reduced compared to those of the wild-type and complemented strains in untreated macrophages (Fig. 2F), as previously shown (Fig. 2A). Interestingly, the number of Δ*orfX* mutant bacteria in macrophages treated with trolox was significantly increased compared to that of Δ*orfX* mutant bacteria in untreated macrophages (Fig. 2F). However, the number of Δ*orfX* mutant bacteria was still significantly reduced compared to those of the wild-type and complemented strains in macrophages treated with trolox (Fig. 2F). Thus, OrfX promotes intracellular survival by dampening the oxidative response of macrophages and, possibly, other mechanisms.

### OrfX is a small secreted protein, whose expression is regulated positively by PrfA.

**FIG 3:**
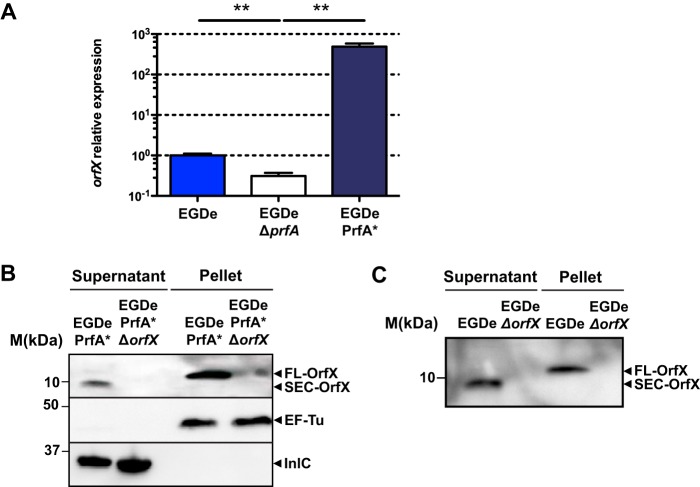
OrfX is a small secreted protein positively regulated by PrfA. (A) Relative expression of OrfX in *L. monocytogenes* EGDe, EGDeΔ*prfA*, and EGDePrfA* after growth in BHI. Relative expression was calculated by qRT-PCR normalized to *rpoB* expression levels. Statistical analysis was performed using the Student two-tailed *t* test. Mean values with *P* values of ≤0.05 were considered statistically significantly different. **, *P* ≤ 0.01. (B and C) Analysis of OrfX production in bacterial culture supernatants and pellets from *L. monocytogenes* EGDePrfA* and EGDePrfA*Δ*orfX* (B) and EGDe and EGDeΔ*orfX* (C) strains. Bacteria were cultured overnight in BHI (B) or BHI-charcoal (C) and harvested. Bacterial pellets were lysed, and culture supernatants were precipitated with 60% ammonium sulfate (B) or trichloroacetic acid (C), dialyzed, and concentrated. Total bacterial extracts and supernatant precipitates were separated by SDS-PAGE. Full-length (FL) and secreted (SEC) OrfX proteins were detected by Western blotting with a rabbit anti-OrfX antiserum. Rabbit anti-EF-Tu antiserum and anti-InlC antiserum were used to control for the presence of EF-Tu in bacterial pellets and InlC in supernatants, respectively. Molecular mass markers are indicated on the left.

The *orfX* gene belongs to the LIPI-1 locus of virulence genes whose expression is induced by PrfA. Additionally, our previous whole-genome transcriptomic analysis suggested that the expression of *orfX* was controlled by PrfA (19). To demonstrate that *orfX* was indeed regulated by PrfA, we compared the levels of its transcription in EGDe, EGDeΔ*prfA*, and EGDePrfA* strains by quantitative reverse transcription-PCR (qRT-PCR). Deletion of PrfA led to decreased levels of the *orfX* transcript compared to its levels in EGDe, while constitutive activation of PrfA led to a massive induction of *orfX* expression (Fig. 3A). Thus, PrfA positively regulates the *orfX* gene, like all the other genes of LIPI-1.

OrfX contains a putative signal peptide covering the first 30 amino acids of its sequence. To address whether *L. monocytogenes* secretes OrfX, we generated polyclonal antibodies against a recombinant OrfX protein and used them to detect the protein in bacterial lysates and culture supernatants. Since the expression of *orfX* is very low in the EGDe strain growing in broth medium but is positively regulated by PrfA, the secretion of OrfX was first investigated using the EGDePrfA* strain. We detected a protein of approximately 15 kDa corresponding to full-length OrfX in bacterial extracts of the wild-type strain but not in those of the EGDePrfA*Δ*orfX* strain (Fig. 3B). We also detected a protein of ≈9 kDa corresponding to leaderless OrfX in culture supernatants of the EGDePrfA* strain after ammonium sulfate precipitation (Fig. 3B). As expected, OrfX could not be detected in culture supernatants of the EGDePrfA*Δ*orfX* strain. For these experiments, InlC, a well-characterized virulence factor secreted by pathogenic *Listeria* species (35, 36), was used as a control (Fig. 3B). The elongation factor EF-Tu, a protein absent from *Listeria* culture supernatant (37), was used as a negative control. Interestingly, OrfX was detected in culture supernatants of the EGDe strain grown in activated-charcoal-treated culture medium, which triggers activation of the PrfA regulon (32) (Fig. 3C). We concluded from these data that OrfX is a new PrfA-regulated virulence factor expressed by *L. monocytogenes* and secreted as a small protein of 9 kDa.

### OrfX interacts with RybP in the nucleus.

Since OrfX is secreted *in vitro* and its expression is induced in infected cells (20), we searched eukaryotic partners of OrfX in order to understand its function in the infection process. We thus performed a yeast two-hybrid screen, using the leaderless OrfX protein as bait and a human placenta library of preys. The RybP protein, which is involved in important processes like development, apoptosis, tumor suppression, and innate immunity (22, 25, 30, 38), was identified as an OrfX interacting partner. The putative region of interaction encompassed the first 178 amino acids of the 228-aa RybP protein.

**FIG 4:**
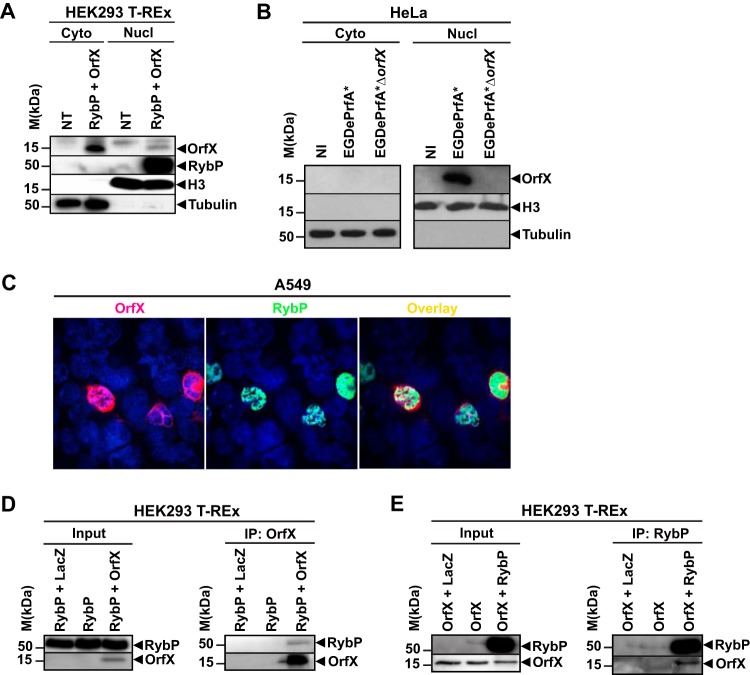
OrfX interacts with RybP in the nucleus. (A to C) OrfX and RybP are detected in the nucleus. (A) Fractionation of HEK-293 T-REx cells either not treated (NT) or transfected with plasmids for OrfX-Myc and RybP-GFP. (B) Fractionation of HEK-293 T-REx cells either not infected (NI) or infected with EGDePrfA* or EGDePrfA*Δ*orfX*. Cytosolic and nuclear fractions were separated by SDS-PAGE. OrfX and RybP were detected by Western blotting with the anti-OrfX antiserum or anti-RybP antibody. Anti-histone H3 and antitubulin antibodies were used to control for the presence of histone H3 in nuclear extracts and α-tubulin in the cytosolic fraction, respectively. Molecular mass markers are indicated on the left. (C) A549 cells were transfected with plasmids for OrfX-Myc and RybP-GFP and visualized by confocal microscopy. OrfX (red) and RybP (green) were detected using anti-OrfX antiserum and anti-RybP antibody, respectively. (D and E) OrfX interacts with RybP. (D) Immunoprecipitation with anti-Myc antibody was performed on lysates of HEK-293 T-REx cells transfected with plasmids for RybP-GFP alone or with LacZ-Myc or OrfX-Myc. Chromatin-enriched fractions (input) or immunoprecipitates (IP) were separated by SDS-PAGE. OrfX and RybP were detected by Western blotting with the anti-OrfX antiserum or anti-RybP antibody. (E) Immunoprecipitation with anti-GFP antibody was performed on lysates of HEK-293 T-REx cells transfected with plasmids for OrfX-Myc alone or with LacZ-Myc or RybP-GFP. Chromatin-enriched fractions or immunoprecipitates were separated by SDS-PAGE. OrfX and RybP were detected by Western blotting with the anti-OrfX antiserum or anti-RybP antibody.

To determine whether RybP and OrfX were located in the same cellular compartments in mammalian cells and also validate the two-hybrid screen, we took a biochemical approach and fractionated cell extracts from transfected or infected cells. HEK-293 T-REx cells expressing OrfX and RybP were processed to separate the cytosolic and nuclear fractions, which were analyzed by immunoblotting using anti-OrfX antiserum and anti-RybP antibody. OrfX was detected in the cytosolic fraction, as well as in the nuclear fraction, while RybP was exclusively detected in the nuclear fraction (Fig. 4A). Histone H3 and α-tubulin were used as nuclear and cytosolic control proteins, respectively. Similarly, HEK-293 T-REx cells infected with EGDePrfA* or EGDePrfA*Δ*orfX* were processed to separate the cytosolic and nuclear fractions, which were analyzed by immmunoblotting using anti-OrfX antiserum. OrfX was exclusively detected in the nuclear fraction of cells infected with EGDePrfA* (Fig. 4B). Histone H3 and α-tubulin were used as the nuclear and cytosolic control protein, respectively.

The subcellular localization of OrfX and RybP was further investigated by confocal fluorescence microscopy of A549 cells expressing OrfX, RybP, or both proteins. OrfX was detected in a heterogeneous nuclear pattern and was also seen in the cytoplasm (Fig. 4C, first panel). As reported (22), RybP was seen in characteristic punctate and larger subnucleolar structures in the nucleoplasm (Fig. 4C, second panel). When OrfX was coexpressed with RybP, the two proteins were found to colocalize to subnucleolar structures (Fig. 4C, third panel).

Additionally, the subcellular localization of OrfX and RybP was determined using split-green fluorescent protein (GFP) complementation. HEK-293 T-REx cells were transfected with RybP-GFP_1–10_ containing the GFP first ten beta sheets, GFP_11_-OrfX containing the GFP eleventh beta sheet, or both recombinant proteins, and the localization of each protein was assessed by fluorescence microscopy. RybP was detected mostly in the nucleus (see Fig. S1, first panel, in the supplemental material). OrfX was detected in both the nucleus and the cytoplasm of transfected cells (Fig. S1, second panel). Fluorescence of the functional reconstituted GFP could only be detected in the nucleus of cells cotransfected with OrfX and RybP (Fig. S1, third panel). An association of GFP_1–10_ with GFP_11_ to form a complete GFP detected as a nuclear signal suggests that protein interaction occurred and that OrfX and RybP could interact in the nuclear compartment.

10.1128/mBio.01550-17.1 FIG S1  OrfX interacts with RybP in the nucleus. Fluorescence images of HEK-293 T-REx cells transfected with plasmids for RybP-GFP_1–10_ and GFP_11_-OrfX are shown. RybP (left) and OrfX (middle) were detected using anti-RybP antibody and anti-OrfX antiserum, respectively. Protein interaction was assessed by detection of GFP fluorescence (right). Download FIG S1, EPS file, 4.2 MB. Copyright © 2017 Prokop et al. 2017 Prokop et al. This content is distributed under the terms of the Creative Commons Attribution 4.0 International license.

To demonstrate further whether OrfX and RybP interact in mammalian cells, we performed immunoprecipitations of RybP-GFP or OrfX tagged with a Myc epitope expressed in HEK-293 T-REx cells. OrfX-RybP interaction was not detected in total cell lysates. Since most RybP is detected in the nucleus (29) and as we have shown that OrfX and RybP are tethered in close proximity in the nucleus (Fig. S1), we hypothesized that OrfX-RybP interaction most likely occurs in the nucleus. We thus performed immunoprecipitations of OrfX-Myc or RybP-GFP from the chromatin-enriched fraction of HEK-293 T-REx cell lysates after Benzonase treatment. Cell lysates were immunoprecipitated with anti-Myc antibody, and precipitates were analyzed by Western blotting using anti-OrfX antiserum and anti-RybP antibody. Myc-tagged OrfX coimmunoprecipitated with RybP-GFP in cells producing both OrfX and RybP but not in cells expressing RybP alone or RybP and LacZ-Myc (Fig. 4D), strongly suggesting an interaction between OrfX and RybP. We next performed the reciprocal coimmunoprecipitation. Cell lysates were immunoprecipitated with anti-GFP antibody, and precipitates were immunoblotted with anti-RybP antibody and anti-OrfX antiserum. RybP-GFP coimmunoprecipitated with OrfX-Myc in cells expressing both proteins but not in cells expressing OrfX-Myc or OrfX-Myc and LacZ-Myc (Fig. 4E), confirming the interaction between OrfX and RybP in mammalian cells.

Taken together, our data reveal that OrfX is a previously unknown bacterial nucleomodulin, i.e., a protein that targets nuclear components of the host cell (39). OrfX is, after LntA (40), the second *Listeria* protein reported to target the nucleus.

### OrfX decreases the protein levels of RybP, which controls cellular infection.

**FIG 5:**
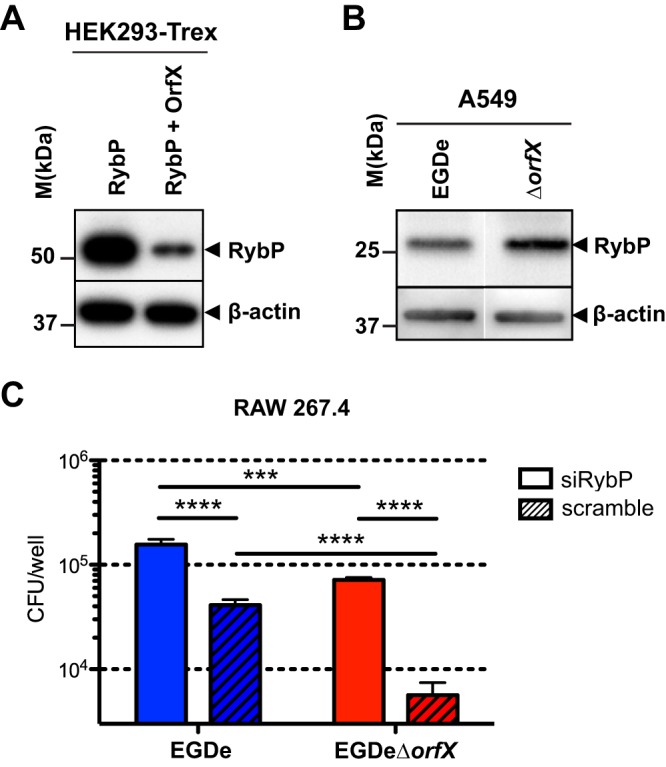
OrfX decreases the levels of RybP, a regulator that protects macrophages upon infection. (A) OrfX reduces the RybP level in transfected cells. HEK-293 T-REx cells were transfected with plasmids for RybP-GFP alone or with OrfX-Myc. After 24 h, cells were lysed. Whole-cell lysates were separated by SDS-PAGE, and Western blots for RybP and β-actin were performed. (B) OrfX reduces the RybP level in infected cells. A549 cells were infected at an MOI of 50 with the wild-type strain (EGDe) or the mutant strain (EGDeΔ*orfX*). After 24 h, cells were lysed. Lysates were separated by SDS-PAGE, and Western blots were performed for RybP and β-actin. (C) RybP decreases *Listeria* survival in macrophages. RybP was knocked down by siRNA transfection (siRybP) of RAW 264.7 macrophages for 24 h, and macrophages were infected with EGDe or EGDeΔ*orfX*. The number of CFU was determined at 12 h after the addition of gentamicin. Statistical analysis was performed using the Student two-tailed *t* test. Mean values are expressed as CFU/well ± SEM. ***, *P* ≤ 0.001; ****, *P* ≤ 0.0001.

To investigate the impact of OrfX’s interaction with RybP, we analyzed the levels of RybP in HEK-293 T-REx cells transfected with RybP alone or cotransfected with OrfX and RybP. Interestingly, the whole-cell protein level of RybP decreased in cells expressing OrfX (Fig. 5A). In contrast, RybP levels remained high in cells overexpressing RybP alone. These data indicate that OrfX negatively regulates the levels of RybP in cells. We next examined RybP in A549 epithelial cells infected with the wild-type EGDe or EGDeΔ*orfX* mutant strain. In agreement with the transfection data, OrfX expression was shown to decrease the level of RybP in infected cells (Fig. 5B).

Having shown that OrfX decreases the level of RybP, we next asked whether OrfX affects some of the known properties of RybP upon infection. Because RybP has the ability to induce cell death (28, 41) and OrfX controls RybP levels, we asked whether OrfX could affect macrophage apoptosis and/or survival. Macrophages were infected with the wild-type EGDe, the EGDeΔ*orfX* mutant, or the complemented strain, and caspase-3 activity (Fig. S2) and lactate dehydrogenase (LDH) release (Fig. S3) were determined 24 h postinfection. The levels of caspase-3 were similar for the three strains, suggesting that OrfX does not modulate the extent of apoptosis of cells infected with *Listeria*. The caspase-3 activator staurosporine, used as a positive control, induced apoptosis of RAW 264.7 cells. The levels of LDH were similar for the three strains, suggesting that OrfX does not trigger necrosis upon infection.

10.1128/mBio.01550-17.2 FIG S2  OrfX does not affect apoptosis. Relative caspase-3 activities of RAW 264.7 cells 24 h after infection. FC, fold change. Cells were either not stimulated (NS), stimulated with 1 mg/ml staurosporine (S), or infected with the wild-type (EGDe), mutant (EGDeΔ*orfX*), or complemented (EGDeΔ*orfX*+*orfX*) strain. Statistical analysis was performed using the Student two-tailed *t* test. Mean values with *P* values of ≤0.05 were considered statistically different. **, *P* ≤ 0.005; ns, nonsignificant difference. Download FIG S2, EPS file, 1.3 MB. Copyright © 2017 Prokop et al. 2017 Prokop et al. This content is distributed under the terms of the Creative Commons Attribution 4.0 International license.

10.1128/mBio.01550-17.3 FIG S3  OrfX does not affect cell viability. LDH release of RAW 264.7 cells 24 h after infection. AU, arbitrary units. Cells were either not stimulated (NS), lysed with water, or infected with the wild-type (EGDe), mutant (EGDeΔ*orfX*), or complemented (EGDeΔ*orfX*+*orfX*) strain. Statistical analysis was performed using the Student two-tailed *t* test. Mean values with *P* values of ≤0.05 were considered statistically different. ****, *P* ≤ 0.0001; ns, nonsignificant difference. Download FIG S3, EPS file, 1.3 MB. Copyright © 2017 Prokop et al. 2017 Prokop et al. This content is distributed under the terms of the Creative Commons Attribution 4.0 International license.

We then addressed whether RybP expression affected the infection of macrophages by *Listeria*. We tested the role of RybP by knocking down its expression with small interfering RNA (siRNA) in macrophages infected with the wild-type EGDe or EGDeΔ*orfX* mutant strain. The number of mutant bacteria was decreased by 1 Log compared to that of wild-type bacteria in cells transfected with the control siRNA (Fig. 5C), confirming the role of OrfX in promoting survival in macrophages (Fig. 2A). Interestingly, the number of mutant bacteria was also decreased but to a lesser extent, approximately 0.5 Log, compared to the number of wild-type bacteria in cells transfected with siRNA against RybP (Fig. 5C), suggesting that OrfX presumably contributes to *Listeria* survival in cells by both RybP-dependent and RybP-independent mechanisms. Strikingly, macrophages transfected with siRNA against RybP showed increased susceptibility to infection with either EGDe or the EGDeΔ*orfX* mutant (Fig. 5C), indicating a protective role for RybP.

Collectively, these results suggest that the main function of OrfX is to contribute to *Listeria* survival in cells by decreasing the level of RybP. Other functions of OrfX, including functions outside the nucleus, remain to be identified.

## DISCUSSION

Here, we report that OrfX is a small protein whose gene is activated by PrfA and secreted by *L. monocytogenes* that plays a critical role in virulence. This virulence factor dampens the oxidative response of macrophages, thereby promoting infection. OrfX is targeted to the cell nucleus, where it interacts with RybP, an important regulator of development, apoptosis, oncogenesis, and innate immunity. The expression of OrfX leads to a decrease in the levels of RybP, contributing to bacterial survival in macrophages. To our knowledge, OrfX is the first microbial protein identified as regulating RybP.

OrfX is a new nucleomodulin. It is the second nuclear effector identified in *Listeria* after LntA, a secreted virulence factor that targets the chromatin repressor BAHD1 to activate interferon-stimulated genes (40). In particular, the interaction of LntA with BAHD1 modulates the lambda interferon (IFN-λ)-dependent immune response to control bacterial colonization of the host (40). Interestingly, OrfX and LntA are structurally unique proteins. They do not bear any detectable protein sequence signatures and do not belong to any of the known nucleomodulin families, such as Ank-, SET- or F-box-containing bacterial proteins. OrfX and many nucleomodulins also lack a classical nuclear localization sequence. How they enter the nucleus remains to be determined. Over the last decade, a growing number of effectors secreted by pathogenic bacteria was found to target the cell nucleus to subvert host defenses (39, 42, 43). Some of these nuclear effectors, such as *Anaplasma phagocytophilum* protein AnkA, interact directly with host DNA to silence the expression of defense genes (44). Others directly target histones. *Chlamydia trachomatis* NUE and *Legionella pneumophila* RomA are methyltransferases that modify histones to repress host gene expression and promote infection (45, 46). Other nucleomodulins associate with nuclear proteins similarly to LntA and OrfX. For example, *Shigella flexneri* OspF and *Salmonella* SpvC are phosphothreonine lyases that dephosphorylate mitogen-activated protein kinases to antagonize the host immune response (47–49). Irreversible dephosphorylation of Erk and p38 by OspF prevents histone H3 phosphorylation and NF-κB-dependent activation of genes involved in innate immune responses (48, 50).

OrfX interacts with RybP, which is primarily found in the nucleus of the cell. RybP is a natively unfolded protein that acquires its structured fold upon binding to DNA or to proteins like the Polycomb H2A monoubiquitin ligases Ring1A and Ring1B (22, 51). RybP is a member of some Polycomb complexes required for maintenance of transcriptional repression during development (22, 52–55). Beyond its requirement at several stages of embryogenesis (56, 57), RybP has been implicated in the induction of apoptosis (26, 28, 41). To our knowledge, the only other virulence factor that has been shown to interact with RybP is apoptin, a small protein from chicken anemia virus that induces apoptosis in transformed and tumor cells (29). However, the outcome of this interaction is unknown. In contrast to apoptin, OrfX does not trigger apoptosis. In the context of *Listeria* infection, OrfX decreases the level of RybP. Interestingly, the negative control of OrfX over RybP seems beneficial for the pathogen, as *Listeria* survived better in macrophages expressing a lower level of RybP. The role of RybP in infection is poorly understood. It has been reported that polymorphism in the human RybP gene is associated with chronic rhinosinusitis (58) and that RybP negatively regulates the Imd immune pathway in *Drosophila* (30). The molecular mechanism of the protective role of RybP in macrophages is currently unknown. It could possibly involve cellular components of host response to infection that are regulated by RybP, such as the tumor suppressor p53 (59–61). An important feature of RybP is its capacity to stabilize p53 (25). The level and activity of p53 are controlled by complex feedback loops involving many regulators whose transcription is activated by p53 (62). A key negative regulator of p53 is the oncogenic protein MDM2 (63). Binding of MDM2 to the N-terminal transactivation domain of p53 inhibits its transcriptional activity. In addition, MDM2 is a RING finger E3 ligase. It ubiquitinates p53 and promotes its degradation. RybP has been shown to interact with MDM2 and decrease MDM2-mediated p53 ubiquitination, leading to increased p53 levels and activity (25). It was recently reported that overexpression of p53 inhibits the invasion of macrophages by *L. monocytogenes*, indicating that the tumor suppressor is required to control *Listeria* infection (64). This study thus supports the hypothesis of an indirect protective role of RybP. Alternatively, the protective role of RybP could depend on its activity at the transcriptional level. However, the genes directly regulated by RybP remain to be characterized.

OrfX dampens superoxide and NO production by infected macrophages, contributing to bacterial survival. Control of reactive oxygen species (ROS) and reactive nitrogen species (RNS) production by OrfX is one of several mechanisms deployed by *Listeria* to avoid intracellular oxidative stress. We previously reported that secretion of the manganese superoxide dismutase (MnSOD) by *Listeria* counteracts the bactericidal oxidative attack of macrophages (65). More recently, it has been shown that *Listeria* prevents the respiratory burst of macrophages by secreting LLO, which inhibits recruitment of the NADPH oxidase NOX2 to the phagosome, thereby restraining the production of ROS (66). The mechanism by which OrfX decreases ROS and RNS production is unknown. OrfX could directly interact with cellular proteins involved in the oxidative response. RybP itself has not been implicated in the control of ROS production so far. However, RybP regulates p53, which has been shown to be involved in the regulation of intracellular ROS and RNS levels (67, 68). Thus, OrfX could also modulate ROS production indirectly by interacting with RybP, and consequently p53.

In conclusion, we have shown that OrfX is a novel PrfA-regulated virulence determinant secreted by *L. monocytogenes* to promote bacterial survival in macrophages by oxidative and nonoxidative pathways. Once secreted, OrfX reaches the nucleus, where it targets RybP. We propose that OrfX modulates the level of RybP to thrive in the intracellular environment while keeping cells alive. Further investigation is now required to fully elucidate OrfX functions inside and outside the nucleus and OrfX synergy with the other virulence factors involved in *Listeria*’s intracellular lifestyle.

## MATERIALS AND METHODS

### Bacterial strains and growth conditions.

*L. monocytogenes* EGDe (BUG1600) and *L. monocytogenes* EGDePrfA* (BUG3057) were used as parental strains (31, 69). *L. monocytogenes* EGDe*ΔorfX* (BUG2160) and *L. monocytogenes* EGDePrfA*-*ΔorfX* (BUG3500) were obtained by gene deletion from *L. monocytogenes* EGDe and *L. monocytogenes* EGDePrfA* as described below. *L. monocytogenes* EGDe/pMK4pPROT (BUG2565), *L. monocytogenes* EGDe*ΔorfX/*pMK4pPROT (BUG1775), *L. monocytogenes* EGDe*ΔorfX/*pMK4pPROT-*orfX* (BUG3134), and *L. monocytogenes* EGDe*ΔorfX/*pIMK2-*orfX* (BUG3603) were obtained by transformation of *L. monocytogenes* EGDe and *L. monocytogenes* EGDe*ΔorfX* as described below. Oligonucleotides used for gene deletion and complementation are listed in Table 1. *L. monocytogenes* EGDe*ΔprfA* (BUG2214) was obtained by gene deletion from *L. monocytogenes* EGDe (19). *Listeria* strains were grown in brain heart infusion (BHI) broth (BD) at 37°C and 200 rpm. *Escherichia coli* strains were grown in lysogeny broth (LB; BD). When required, chloramphenicol and erythromycin were used at final concentrations of 7 and 5 μg/ml for *Listeria*. Ampicillin and kanamycin were used at final concentrations of 100 and 50 μg/ml for *E. coli*.

**TABLE 1:** Oligonucleotides used in this study

Name	Sequence (5′→3′) and restriction site^a^	Enzyme	Use
Lmo0206-1	TGT**ACGCGT**TCGGTGACTGATTACCGA	MluI	*orfX* deletion (pMAD)
Lmo0206-2	GCTT**GAATTC**TTGGCTTACTTCCTCCCC	EcoRI	
Lmo0206-3	TGG**GAATTC**AGTTACGATGAGTTGTGACT	EcoRI	
Lmo0206-4	TGG**CCATGG**TCATATCTTCCGAAGAGATTTTAG	NcoI	
Lmo0206-5	ATGTATATAAAAGGGAGGTTAATC		
Lmo0206-6	ACTCTTCGTTCGCAATCC		
U415	CATG**CCATGG**GCTCCGTATTAATATTGCTTATA	NcoI	OrfX expression (pET28b)
L416	GGTG**CTCGAG**CTCTTCGTTCGCAATCCC	XhoI	
U418	CGC**GGATCC**ATGTATATAAAAGGGAGGTTAATC	BamHI	*orfX* complementation (pMK4)
L419	GGTG**CTGCAG**TTACTCTTCGTTCGCAATCCC	PstI	
U417	CATG**CCATGG**GCTATATAAAAGGGAGG	NcoI	*orfX* complementation (pIMK2)
L418	AATG**CCATGG**TTACTCTTCGTTCGCAATCC	NcoI	
U435	ATAT**GCGGCCGC**CGTTTCGAAAGGCGAG	NotI	Split GFP (GFP_1–10_)
L436	CC**TCTAGA**CCTTTCTCGTTTGGGTCTTTGCTCAGC	XbaI	
U437	AA**GGTACC**GAGGAGATCTGCCGCCGCG	KpnI	Split GFP (RybP)
L438	ATAT**GCGGCCGC**GAAAGATTCATCATTGAC	NotI	
U439	CC**GGTACC**ATGGGCCGGGACCACATGG	KpnI	Split GFP (GFP_11_)
L440	TATA**GCGGCCGC**CGGATCCGCTGCCG	NotI	
U441	TATA**GCGGCCGC**TCTGTCCTCATCC	NotI	Split GFP (OrfX′)
L442	GC**TCTAGA**CTCCTCGTTAGCAATGCCCTTC	XbaI	
qPCR-orfX-for	ATG TAT ATA AAA GGG AGG TTA ATC TTT TTC		qRT-PCR
qPCR-orfX-rev	CGC TTA AAT GAA ATG GTT CAT CTT TC		
qPCR-gyrA-for	TCGGCATGGAAGTACTGGAG		
qPCR-gyrA-rev	ACACCCATACCACCACGATT		
qPCR-rpoB-for	GCGAACATGCAACGTCAAGCAGTA		
qPCR-rpoB-rev	ATGTTTGGCAGTTACAGCAGCACC		

^a^ Boldface indicates a restriction site.

### *L. monocytogenes* Δ*orfX* mutant strains.

The 500-bp upstream and 490-bp downstream regions flanking the *orfX* gene (*lmo0206*) were amplified by PCR using chromosomal DNA from *L. monocytogenes* EGDe and the oligonucleotides Lmo0206-1/Lmo0206-2 and Lmo0206-3/Lmo0206-4, respectively (Table 1). The upstream MluI-EcoRI- and downstream EcoRI-NcoI-restricted fragments were cloned sequentially into the thermosensitive plasmid pMAD (70), constructing pMAD-0206. This plasmid was electroporated into *L. monocytogenes* EGDe or *L. monocytogenes* EGDePrfA* at 2,500 V, 250 Ω, and 25 μF. Generation of the *orfX* deletion mutation by allelic exchange was carried out as described previously (70). The *ΔorfX* mutants were identified by PCR on colonies plated on BHI–X-Gal (5-bromo-4-chloro-3-indolyl-β-d-galactopyranoside) using oligonucleotides Lmo0206-5/Lmo0206-6 (Table 1). The deletion of *orfX* was checked by sequencing.

### *L. monocytogenes* complementation strains.

The *orfX* gene was amplified by PCR using chromosomal DNA from *L. monocytogenes* EGDe and oligonucleotides U418/L419 or U417/L418 (Table 1). The BamHI/PstI and NcoI PCR products were cloned into replicative plasmid pMK4pPROT (37) or integrative plasmid pIMK2 (71), respectively. The presence of the *orfX* gene was confirmed by PCR amplification and sequencing. The resulting plasmids, pMK4pPROT-*orfX* and pIMK2-*orfX*, were used to electroporate *L. monocytogenes* EGDe*ΔorfX* at 2,500 V, 250 Ω, and 25 μF, generating *L. monocytogenes* EGDe*ΔorfX/*pMK4pPROT-*orfX* and *L. monocytogenes* EGDe*ΔorfX/*pIMK2-*orfX*, respectively. The expression of *orfX* was confirmed by performing RT-PCR and Western blotting.

### Animal studies.

*L. monocytogenes* was thawed from glycerol stocks stored at −80°C, washed, and diluted in phosphate-buffered saline (PBS) before injection. A sublethal dose (10^4^ *L. monocytogenes*) was injected into the lateral vein of the tail of 8-week-old female BALB/c mice (Charles River, Inc.). The inoculum was confirmed by plating serial dilutions of the bacterial suspension on BHI agar plates. For determination of bacterial loads, livers and spleens were recovered and disrupted in PBS at 24, 48, and 72 h postinfection. Serial dilutions of organ homogenates were plated on BHI agar plates, and CFU were counted after growth at 37°C for 48 h. All experiments were performed in accordance with the Institut Pasteur’s guidelines for laboratory animal welfare.

### Cell lines and culture media.

The human kidney cell line HEK-293 T-REx stably expressing the tetracycline repressor (Invitrogen) was cultured in Dulbecco’s modified Eagle’s medium (DMEM; Gibco) supplemented with 10% tetracycline-free fetal bovine serum (FBS; BioWest), 2 mM l-glutamine (Gibco), and 5 μg/ml blasticidin at 37°C in 10% CO_2_ atmosphere. The murine macrophage-like cell line RAW 264.7 (ATCC TIB-71) was cultured in DMEM (Gibco) supplemented with 10% FBS (BioWest) at 37°C in 10% CO_2_ atmosphere. The human cervical cell line HeLa (ATCC CCL-2) was cultured in modified Eagle’s medium (MEM; Gibco) supplemented with 10% FBS (BioWest), 1 mM sodium pyruvate (Gibco), and 0.1 mM nonessential amino acids (Gibco) at 37°C in 10% CO_2_ atmosphere. The human pulmonary cell line A549 (ATCC CCL-185) was cultured in F-12K medium (Gibco) supplemented with 10% FBS (BioWest) at 37°C in 10% CO_2_ atmosphere.

### Cell infection.

RAW 264.7 macrophages were seeded onto 6-well, 12-well, or 48-well plates at a density of 5 × 10^5^ or 2 × 10^5^ cells per well 24 h before infection. When required, cells were incubated in complete medium containing 50 μM trolox (Sigma). Cells were infected with *L. monocytogenes* strains at a multiplicity of infection (MOI) of 10:1 or 20:1 in serum-free medium and either incubated at 37°C for 1 h or centrifuged at 300 × *g* for 2 min and incubated at 37°C for 15 min to allow phagocytosis. Nonphagocytosed bacteria were washed away with PBS, and 20 µg/ml gentamicin-containing medium was added to prevent the growth of extracellular bacteria. The number of intracellular bacteria, expression of cellular proteins, and production of NO were assessed as described in other sections.

HeLa cells were infected with *L. monocytogenes* strains at an MOI of 20:1. After 1 h of infection, cells were washed in PBS and treated with 20 µg/ml gentamicin to prevent the growth of extracellular bacteria. The expression of cellular proteins was assessed as described in other sections.

A549 cells were infected with *L. monocytogenes* strains at an MOI of 50:1. After 1 h of infection, cells were washed in PBS and treated with 20 µg/ml gentamicin to prevent the growth of extracellular bacteria. After 24 h, the expression of cellular proteins was assessed as described in other sections.

### DHE staining.

RAW 264.7 macrophages were cultured on glass coverslips in 24-well plates in complete medium. Macrophages were infected with *Listeria* strains as described above. Cells were washed once with PBS and stained for 45 min with 5 µM dihydroethidium (DHE; Sigma) in PBS. Cells were washed and fixed with 4% paraformaldehyde (PFA) in PBS. Samples were mounted on glass coverslips with Fluoromount medium (Electron Microscopy Sciences) and were observed with a Zeiss Axiovert 200 M epifluorescence microscope (Carl Zeiss, Inc.) connected to a charge-coupled device (CCD) camera, using DAPI (4′,6-diamidino-2-phenylindole) and rhodamine filters. Images were acquired with an apochromat 63× oil immersion objective (Carl Zeiss, Inc.) and processed with Metamorph software (Universal Imaging).

### Griess assay.

RAW 264.7 macrophages were infected as described above or treated with 1 µg/ml LPS for 24 h. Nitric oxide production by macrophages was measured by the Griess reaction using a Griess assay kit (Promega) according to the manufacturer’s protocol. The absorbance was measured at 530 nm on a microplate reader (Berthold). Data were normalized against the absorbance of the culture medium, and the nitrite concentration was determined using the standard curve.

### qRT-PCR.

*L. monocytogenes* was grown in BHI at 37°C under constant agitation until reaching an optical density at 600 nm (OD_600_) of 0.9, pelleted, and frozen at −80°C. RNAs were extracted as previously described (72). For each sample, 10 µg of RNA was treated with DNase I (Turbo DNA-free kit, Ambion). RNAs (500 ng) were reverse transcribed with Quantiscript reverse transcriptase (QuantiTect reverse transcription [RT] kit; Qiagen). Quantitative RT-PCRs (qRT-PCRs) were carried out using oligonucleotides qPCR-orfX-for, qPCR-orfX-rev, qPCR-gyrA-for, qPCR-gyrA-rev, qPCR-rpoB-for, and qPCR-rpoB-rev (Table 1), and the products quantified with SYBR green master mix on a C1000 Touch CFX384 machine (Bio-Rad). The expression levels of *orfX* were normalized to those of *L. monocytogenes rpoB* and *gyrA* genes, and the fold changes in expression were calculated using the cycle threshold (ΔΔ*CT*) method. All samples were evaluated in triplicate and in at least three independent experiments.

### Recombinant proteins and antibodies.

The coding region of *orfX*, excluding the secretion signal sequence, was amplified by PCR using chromosomal DNA from *L. monocytogenes* EGDe and the oligonucleotides U415 and L416 (Table 1). The PCR product was digested by NcoI and XhoI and cloned into the expression vector pET-28b (Novagen), generating pET-28b-OrfX-6His, which was maintained in *E. coli* strain TOP10 (BUG2930). The presence of the gene encoding OrfX-6His was confirmed by PCR amplification and sequencing. *E. coli* strain Rosetta was transformed with pET-28b-OrfX-6His and grown in 4,200 ml autoinducing medium ZYP5052 at 37°C to an *A*_600_ of 1.1 and then at 17°C to an *A*_600_ of 10. The bacteria were harvested, and the pellet was resuspended in 400 ml of lysis buffer (50 mM Tris, 300 nM NaCl, 10 mM imidazole, pH 8.0, 0.25 mg/ml lysozyme, 1 mM phenylmethylsulfonyl fluoride [PMSF]). The recombinant OrfX was purified on a HisTrap nickel column. Rabbit polyclonal anti-OrfX antibodies (R218 and R219) were generated against OrfX-6His as described previously (37). Anti-OrfX antibodies were purified by affinity on a Sepharose column as described by Kocks et al. (73).

The primary antibodies used in this study were mouse monoclonal anti-T7 (Novagen), rabbit polyclonal anti-TurboGFP (Evrogen), mouse monoclonal anti-c-Myc (9E10; Santa Cruz), mouse polyclonal anti-human RybP (Sigma), mouse monoclonal anti-actin (AC-15; Sigma), rabbit polyclonal anti-histone H3 (9715; Cell Signaling), mouse monoclonal anti-α-tubulin (T6074, Sigma), mouse monoclonal anti-lamin A/C (4777; Cell Signaling), rabbit polyclonal anti-EF-Tu (R114) (37), and rabbit polyclonal anti-InlC (R134) (74) antibodies. The secondary antibodies were Alexa Fluor 350- and 546-conjugated goat anti-mouse and goat anti-rabbit antibody, respectively (Molecular Probes) and horseradish peroxidase (HRP)-conjugated goat anti-mouse and goat anti-rabbit (AbCys) antibodies.

### Preparation of total bacterial extracts and supernatant precipitation.

*L. monocytogenes* strains were cultured at 37°C in 10 ml BHI or BHI supplemented with 0.2% charcoal (Merck). Bacterial pellets were recovered after centrifugation at 10,000 rpm for 10 min, washed twice in PBS, and resuspended in 100 µl of B-PER II bacterial protein extraction reagent (Pierce) to isolate total bacterial proteins. Culture supernatants were precipitated in 20, 40, 60, and 80% ammonium sulfate or trichloroacetic acid at 4°C overnight. After centrifugation, pellets were washed twice with 5 ml cold acetone, dried, and resuspended in 200 µl of 2× Laemmli buffer.

### Western blotting.

Samples were separated by SDS-PAGE and transferred onto a polyvinylidene difluoride (PVDF) Hybond P membrane (Amersham) by semidry blotting at 2.5 V for 8 min (Fast-blotter; Invitrogen). The membranes were blocked in 5% blotto overnight at 4°C and probed with the primary antibody in 5% blotto for 3 h at room temperature. After incubation with goat anti-rabbit or anti-mouse IgG horseradish peroxidase-conjugated secondary antibody diluted at 1:8,000 in 0.1% Tween–PBS for 1 h at room temperature, the blots were revealed using the ECL or ECL-2 kit (Thermo Fisher Scientific).

### Two-hybrid screen.

The *orfX* gene without the secretion signal sequence (encoding amino acids 25 to 107) was amplified by PCR using chromosomal DNA from *L. monocytogenes* EGDe. The PCR product was cloned in frame with the LexA DNA binding domain into the bait plasmid pB27 (Hybrigenics). The prey plasmid derived from pGADGH was constructed using the human placenta RP4 library. The two-hybrid screen was performed by Hybrigenics using the ULTImate Y2H technique. Prey fragments were amplified by PCR and sequenced.

### Plasmids.

Plasmids were prepared as described previously (37). The mammalian cell expression plasmids were pCMV6/RybP-GFP (OriGene), pcDNA4/TO/*myc*-His/*lacZ* (Life Technologies, Inc.), and pcDNA4/TO/*myc*-His/*orfX′*, containing an *orfX* sequence that has been optimized for expression in mammalian cells (Genecust).

The split-GFP plasmids were pcDNA4/TO/GFP_11_-OrfX′/*myc*-HisA and pcDNA4/RybP-GFP_1–10_/V5-HisB. Briefly, human *rybP* without the stop codon was amplified by PCR from pCMV6/RybP-GFP using oligonucleotides U437/L438 (Table 1). The PCR product was cloned into pCR-Blunt II-TOPO (Invitrogen, Life Technologies, Inc.), generating pOD103. The sequence of *gfp*_1–10_, except for the start and stop codons, was amplified by PCR from pLVX-GFP_1–10_, a gift from Matteo Bonazzi (CBPS, Montpellier, France), using oligonucleotides U435/L436. The PCR product was cloned into pCR-Blunt II-TOPO, generating pOD104. The sequence of *gfp*_1*–*10_ from pOD104 was subcloned into NotI/XbaI pcDNA4/V5-HisB (Invitrogen, Life Technologies, Inc.), constructing pOD107. The *rybP* gene from pOD103 was subcloned into KpnI/NotI pOD107, creating pOD108. The plasmid pOD108 (pcDNA4/RybP-GFP_1–10_/V5-HisB) was maintained in *E. coli* TOP10 (BUG3430). The sequence of *gfp*_11_ without the stop codon was amplified by PCR from pLVX-GFP_11_-Rab5, a gift from Matteo Bonazzi (CBPS, Montpellier, France), using oligonucleotides U439/L440. The PCR product was cloned into pCR-Blunt II-TOPO, generating pOD105. The *orfX′* gene was optimized for expression in mammalian cells. It was amplified without start and stop codons by PCR from pcDNA4/TO/*myc*-His/*orfX′* (Genecust) using oligonucleotides U441/L442. The PCR product was cloned into pCR-Blunt II-TOPO, generating pOD106. The *orfX′* gene from pOD106 was subcloned into NotI/XbaI pcDNA4/TO/*myc*-HisA, creating pOD109. The sequence of *gfp*_11_ from pOD105 was subcloned into KpnI/NotI pOD109, constructing pOD110. The plasmid pOD110 (pcDNA4/TO/GFP_11_-OrfX′/*myc*-HisA) was maintained in *E. coli* TOP10 (BUG3432).

### Cell fractionation.

HEK-293 T-REx cells were cultured in T75 flasks in complete medium containing 5 µg/ml blasticidin. Cells were transfected with 10 µg of pCMV6/RybP-GFP, pcDNA4/TO/*myc*-His/*lacZ*, or pcDNA4/TO/*myc*-His/*orfX′* in Lipofectamine 2000 according to the manufacturer’s protocol (Invitrogen). After 24 h of transfection, expression was induced with 1 µg/ml doxycycline. After 6 h, cells were pelleted at 4°C.

HeLa cells cultured in 100-mm dishes were infected with *L. monocytogenes* strains at an MOI of 20:1. After 1 h of infection, cells were washed in PBS and treated with 20 µg/ml gentamicin to prevent the growth of extracellular bacteria. After 18 h of infection, cells were pelleted at 4°C.

Transfected or infected cell pellets were resuspended in 3 volumes of buffer A (20 mM HEPES, pH 7, 0.15 mM EDTA, 0.15 mM EGTA, 10 mM KCl) supplemented with 1× PhosSTOP (Roche), 1× Complete EDTA-free (Roche), 0.5 mM spermine, and 0.15 mM spermidine at 4°C. Cells were lysed in 1% (vol/vol) NP-40 (Igepal CA-630) by vortexing for 20 s at 4°C. Lysates were mixed with 8/9 volumes of buffer SR (50 mM HEPES, pH 7, 0.25 mM EDTA, 10 mM KCl, 70% [wt/vol] sucrose) supplemented with 1× PhosSTOP, 1 × Complete EDTA-free, 0.5 mM spermine, and 0.15 mM spermidine at 4°C. Samples were centrifuged for 5 min at 2,000 × *g* to recover the insoluble fraction, which contains nuclei. Supernatants were centrifuged for 20 min at 20,000 × *g* to eliminate cell debris, and the new supernatants, corresponding to cytosolic extracts, were collected. Nuclei were washed in 3 volumes of buffer B (10 mM HEPES, pH 7, 0.1 mM EDTA, 100 mM NaCl, 25% [wt/vol] glycerol) supplemented with 1× PhosSTOP, 1× Complete EDTA-free, 0.5 mM spermine, and 0.15 mM spermidine at 4°C. Samples were centrifuged for 5 min at 2,000 × *g*. Pellets were resuspended in 2 volumes of sucrose buffer (20 mM Tris, pH 7.65, 60 mM NaCl, 15 mM KCl, 0.34 M sucrose) supplemented with 1× PhosSTOP, 1× Complete EDTA-free, 0.5 mM spermine, and 0.15 mM spermidine at 4°C. High-salt buffer (20 mM Tris, pH 7.65, 0.2 mM EDTA, 25% glycerol, 900 mM NaCl, 1.5 mM MgCl_2_) supplemented with 1× PhosSTOP, 1× Complete EDTA-free, 0.5 mM spermine, and 0.15 mM spermidine was added drop by drop to reach a final concentration of 300 nM. Samples were incubated on ice for 30 min and homogenized every 5 min. Sucrose buffer (1/3 vol) was added, and samples were centrifuged for 10 min at 10,000 × *g*. Supernatants corresponding to soluble nuclear fractions were collected. Pellets were resuspended in 2 volumes of sucrose buffer. Concentrations of 0.0025 U/μl micrococcal nuclease and 1 mM CaCl_2_ were added to the samples, which were incubated for 15 min at 37°C. After the addition of 4 mM EDTA, samples were sonicated using a Bioruptor (Diagenode) for 30 s every minute for 10 min and centrifuged for 15 min at 13,000 × *g*. Supernatants corresponding to the chromatin fractions were collected. Samples were separated by SDS-PAGE on 4 to 15% Mini-Protean TGX stain-free polyacrylamide gels (Bio-Rad) and subjected to Western blot analysis.

### Fluorescence assay.

A549 cells were cultured on glass coverslips in 24-well plates. Cells were transfected with 10 µg of pcDNA4/TO/*myc*-His/*orfX′* and/or pCMV6/RybP-GFP in Lipofectamine 2000 according to the manufacturer’s protocol (Invitrogen). After 24 h of transfection, cells were fixed for 20 min at room temperature with 4% paraformaldehyde in PBS, permeabilized with 0.5% Triton X-100 in PBS for 4 min at room temperature, and blocked in 1% bovine serum albumin (BSA)–PBS.

HEK-293 T-REx cells were cultured on glass coverslips in 24-well plates in complete medium containing 5 µg/ml blasticidin. Cells were transfected with 10 µg of pcDNA4/TO/GFP_11_-OrfX′/*myc*-HisA and/or pcDNA4/RybP-GFP_1–10_/V5-HisB in Lipofectamine 2000 according to the manufacturer’s protocol (Invitrogen). After 24 h of transfection, expression was induced with 1 µg/ml doxycycline. After 6 h, cells were fixed for 20 min at room temperature with 4% paraformaldehyde in PBS, permeabilized with 0.5% Triton X-100 in PBS for 4 min at room temperature, and blocked in 1% BSA–PBS.

Fixed cells were incubated with anti-RybP or anti-OrfX primary antibodies for 1 h and then with Alexa Fluor 350-conjugated anti-mouse or Alexa Fluor 546-conjugated anti-rabbit secondary antibody, respectively, for 30 min. Samples were mounted on glass coverslips with Fluoromount medium (Electron Microscopy Sciences) and were observed with a Zeiss Axiovert 200 M epifluorescence microscope (Carl Zeiss, Inc.) connected to a CCD camera. Images were acquired with an apochromat 63× oil immersion objective (Carl Zeiss, Inc.) and processed with Metamorph software (Universal Imaging).

### Protein immunoprecipitation.

HEK-293 T-REx cells were cultured in T75 flasks in complete medium containing 5 µg/ml blasticidin. Cells were transfected with 10 µg of pCMV6/RybP-GFP, pcDNA4/TO/*myc*-His/*lacZ*, or pcDNA4/TO/*myc*-His/*orfX′* in Lipofectamine 2000 according to the manufacturer’s protocol (Invitrogen). After 24 h of transfection, expression was induced with 1 µg/ml doxycycline. After 6 h, cells were washed once with cold PBS and lysed in NP-40 lysis buffer consisting of 50 mM Tris-HCl, pH 7.5, 150 mM NaCl, 1% NP-40, 0.25% deoxycholic acid, 1× protease cocktail (Roche-EDTA), 1 mM AEBSF [4-(2-aminoethyl)benzenesulfonyl fluoride hydrochloride], 1× PhosSTOP (Roche), 2 mM MgCl_2_, and 2.5 µl/ml Benzonase (Novagen). Lysis was performed for 1 h at 4°C, followed by 15 min of incubation at 37°C. Two millimolars EDTA was added to the lysates. Samples were centrifuged for 1 h at 13,000 rpm at 4°C. Supernatants were collected and incubated for 2 h with 50 µl of 50% protein A–Sepharose CL slurry (GE Healthcare). Samples were centrifuged for 1 min at 1,000 rpm, incubated overnight at 4°C with 25 µg of antibodies directed against the protein to be immunoprecipitated, and treated for 3 h with 50 µl of 50% protein A–Sepharose CL slurry. The protein A beads were washed three times in lysis buffer without MgCl_2_, and proteins were eluted with 30 µl of hot 2× Laemmli buffer. Samples were separated by SDS-PAGE on 10% polyacrylamide gels and subjected to Western blot analysis.

### Caspase 3 and LDH assays.

RAW 264.7 cells were either not stimulated, stimulated with 1 μg/ml staurosporine, or infected with the wild-type (EGDe), mutant (EGDeΔ*orfX*), or complemented (EGDeΔ*orfX*+*orfX*) strain for 24 h. Caspase 3 is an effector caspase critical for apoptosis. Caspase 3 activity of RAW 264.7 cell lysates was measured by using a caspase 3 colorimetric assay (Sigma) according to the manufacturer’s recommendations.

RAW 264.7 cells were either not stimulated, lysed with water, or infected with the wild-type (EGDe), mutant (EGDeΔ*orfX*), or complemented (EGDeΔ*orfX*+*orfX*) strain for 24 h. Cell lysis was measured by using the Cytotox 96 nonradioactive cytotoxicity assay (Promega), based on the detection of LDH released in culture supernatants, according to the manufacturer’s recommendations.

### Electroporation of macrophages.

RAW 264.7 macrophages were cultured in 12-well plates and electroporated with 400 nM siRNA against mouse RybP or 400 nM scrambled siRNA using the Cell Line Nucleofactor kit C and the Nucleofector 2b device program version X-005 (Lonza). After 24 h, cells were infected as described above. Cells were lysed for 15 min in 0.2% Triton X-100 12 h postinfection, and lysates were plated on BHI plates. Plates were incubated at 37°C for 24 h, and CFU counted.

### Statistical analysis.

All experiments were performed independently and repeated two to six times. *P* values of ≤0.05 were considered statistically significant in Student’s *t* test. Data are represented as the mean values ± standard errors of the means (SEM).

### Ethics statement.

This study was carried out in strict accordance with the French national and European laws and conformed to the Council Directive on the approximation of laws, regulations, and administrative provisions of the Member States regarding the protection of animals used for experimental and other scientific purposes (86/609/EEC [75]). Experiments that relied on laboratory animals were performed in strict accordance with the Institut Pasteur’s regulations for animal care and use protocol, which was approved by the Animal Experiment Committee of the Institut Pasteur (approval number 03-49).
